# Vibration Energy Harvesting by Means of Piezoelectric Patches: Application to Aircrafts

**DOI:** 10.3390/s22010363

**Published:** 2022-01-04

**Authors:** Domenico Tommasino, Federico Moro, Bruno Bernay, Thibault De Lumley Woodyear, Enrique de Pablo Corona, Alberto Doria

**Affiliations:** 1 Department of Industrial Engineering, University of Padova, 35131 Padova, Italy; federico.moro@unipd.it (F.M.); alberto.doria@unipd.it (A.D.); 2 SONACA, SA, 6041 Gosselies, Belgium; Bruno.BERNAY@sonaca.com (B.B.); Thibault.DELUMLEYWOODYEAR@sonaca.com (T.D.L.W.); 3 Smart Material Corp., 01159 Dresden, Germany; e.depablo@smart-material.com

**Keywords:** vibration energy harvesting, slat vibrations, multi-physics model, piezoelectric harvester

## Abstract

Vibration energy harvesters in industrial applications usually take the form of cantilever oscillators covered by a layer of piezoelectric material and exploit the resonance phenomenon to improve the generated power. In many aeronautical applications, the installation of cantilever harvesters is not possible owing to the lack of room and/or safety and durability requirements. In these cases, strain piezoelectric harvesters can be adopted, which directly exploit the strain of a vibrating aeronautic component. In this research, a mathematical model of a vibrating slat is developed with the modal superposition approach and is coupled with the model of a piezo-electric patch directly bonded to the slat. The coupled model makes it possible to calculate the power generated by the strain harvester in the presence of the broad-band excitation typical of the aeronautic environment. The optimal position of the piezoelectric patch along the slat length is discussed in relation with the modes of vibration of the slat. Finally, the performance of the strain piezoelectric harvester is compared with the one of a cantilever harvester tuned to the frequency of the most excited slat mode.

## 1. Introduction

Vibration energy harvesters equipped with piezoelectric materials have been subject of research for decades both in the private and the public sectors. These materials’ applications towards energy harvesting, however, are now experiencing a bigger boom thanks to the opportunities offered through low-power sensing systems [1,2,3] coupled with modern wireless data-transfer modules [4,5]. Thanks to the aforementioned technologies and the ever-increasing IoT applications within the industry, piezoelectric harvesters have proved to be especially useful in cases where strong constraints such as allowed weight, space availability, low maintenance components, temperature fluctuations, extreme pressures among others are present within the system. Piezoelectric patches in the form of thin films can be embedded onto vibrating structures at hard-to-reach places, where other forms of energy sources (such as batteries) are not feasible to mount on. These patches can be previously fine-tuned (through different means such as simulation of patch-geometry or cantilever structures) to have a natural frequency within the range of the vibrating frequency of the structure, allowing it to further improve energy production. Current piezoelectric harvesters are based on materials that contain toxic elements (Pb, Bi, Te, Sb) or rare earth elements. Therefore, the widespread implementation of harvesting technologies is still difficult, and research efforts are made in order to develop eco-friendly and cost-efficient piezoelectric materials [6].

The most important use of piezoelectric harvesters in aeronautics is as a power supply of structural health monitoring (SHM) systems, i.e., the process of damage detection in machinery and structures [3,7,8]. In particular, in the last years there has been a great deal of interest in developing new technologies for autonomous SHM systems (avoiding additional cabling), consisting of embedded sensors, data acquisition, wireless communication and energy harvesters. The harvesting system can take the form either of a cantilever oscillator covered by a piezoelectric layer or of a piezo-patch bonded to the host structure:

- Cantilever harvesters exploit the resonance phenomenon and are usually equipped with a tip mass for tuning to the vibrations of the host structure (see, e.g., [9,10]). Even though resonance can greatly improve the harvester performance, it increases the stress in the piezo material with the risk of fatigue failure and can cause safety issues if aircraft structure is light;
- Piezoelectric patches are attached directly to the host structure and directly exploit the strain of the host structure caused by vibration (see, e.g., [11,12,13]). They are well suited for aeronautic applications since they are lightweight (i.e., no need of tip mass) and do not operate in resonance condition.

**Figure 1:**
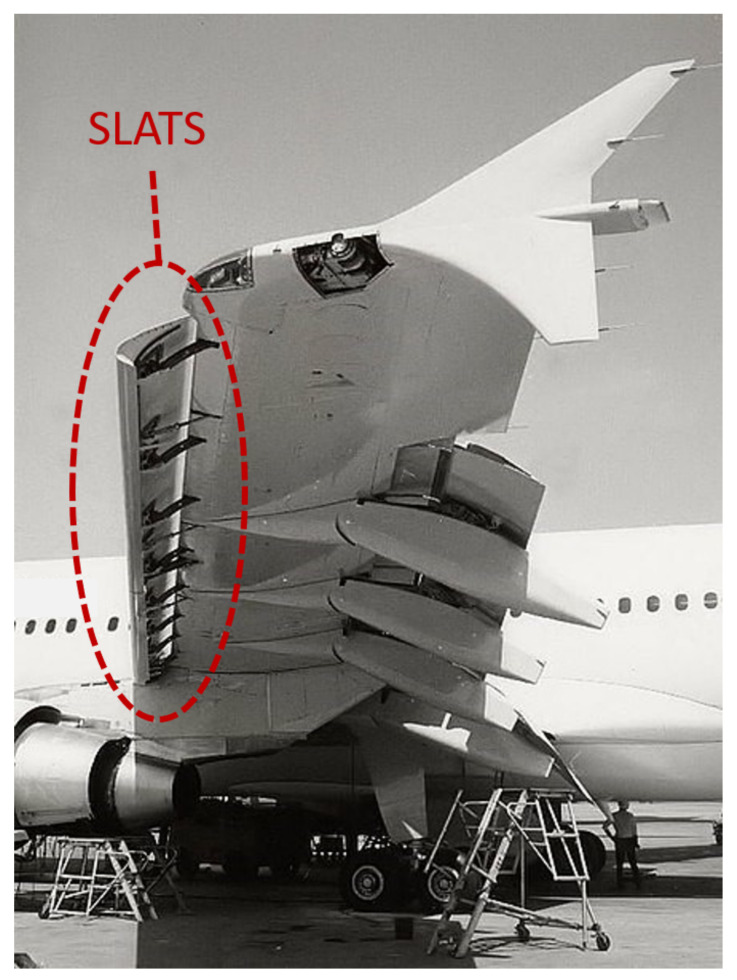
Wing with slats.

The highest amount of energy is extracted when the patch is placed in correspondence of a vibration anti-node of a strongly excited mode of vibration of the host structure. The optimization process [14] for finding the best patch position on the wing is rather difficult, since aircraft elements typically have many complex modes of vibration that can be excited at the same time by broad-band excitation [15]. The slat is a wing component that is excited by a large amount of vibration and that has been scarcely considered in the literature in vibration energy harvesting applications (for instance, in [11] the slat mass is distributed on the whole spar but is not considered alone). The slat is a movable component of the wing leading edge which increases the lift of the wing when it is deployed, see Figure 1.

The vibrations widely vary depending on the type of aircraft (airliners, business jet, cargo jet, etc.), type of engine, type of flight (long/short range). Even in normal operating conditions, there are different vibrations sources for the slats, such as:

- Vibrations from the engines, which are generally defined as random because the frequency and the magnitude vary with the time. They are present along the entire flight;
- Vibrations due to the movement of the slats when they are deployed or retracted for take-off and landing phases. This type of vibrations is generated with high frequencies and magnitude but is limited to the duration of the operation. Such vibrations are predictable because slats are actuated at a well-known step of the flight;
- Random vibrations caused by the turbulence. The frequencies and magnitudes can widely vary and are not fully predictable even if they are considered as “normal” for most flights;
- Vibrations caused by on-ground maneuvers such as runway bumps during takeoff or landing.

Random vibrations due to the engine and turbulence are the most exploitable, since they are present for the entire duration of the flight both when the slat is deployed and when it is retracted. Basic studies about harvesting from random vibrations were carried out in [16,17], where the power spectral density (PSD) of the harvester output voltage was related to the PSD of random vibrations by an equivalent lumped-element electromechanical model. The problem of energy harvesting from random vibration of thin structures by means of multiple piezo-patches was addressed in [18] both by numerical and experimental investigations.

The present paper is organized as follows. In Section 2, a semi-analytical model of a vibrating slat is presented. In Section 3, the calculation of voltage and power generated by a piezoelectric (PE) patch is carried out and numerical results showing the effect of the position of the PE patch on the slat are presented. In Section 4, the performance of a cantilever harvester mounted on the slat and equipped with the same piezoelectric layer of Section 3 is analyzed. The last sections deal with the comparison between these different harvester arrangements.

## 2. Mathematical Model of the Vibrating Slat

**Figure 2:**
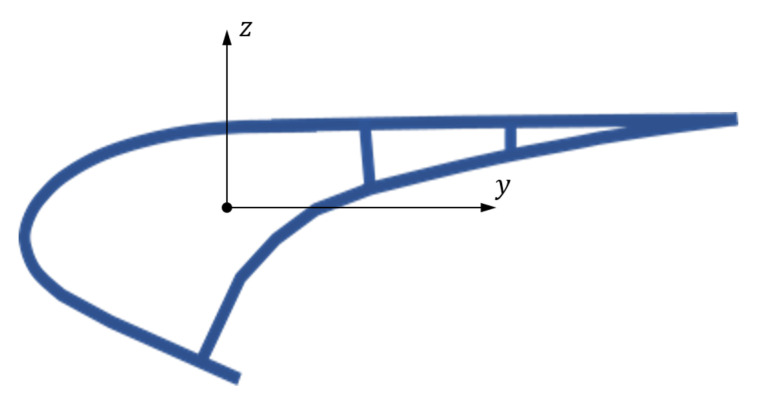
Cross-section of the slat.

The slat here considered is built by SONACA, has the cross-section represented in Figure 2 and is made of composite material.

**Figure 3:**
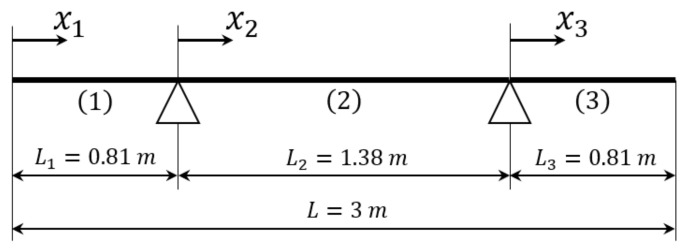
Geometrical scheme of the deployed slat. The slat is divided in three parts with length $L_{i}$ and local reference frame $x_{i}$, i=1,2,3.

The deployment and retraction of the slat is allowed by two servomechanisms that are connected to two sections of the slat. Therefore, the whole deployed slat can be considered a pinned beam with overhangs, see Figure 3, which also gives the main dimensions of the slat.

### 2.1. Modes of Vibration

The slat is considered a Euler–Bernoulli beam with constant cross-section. The parameters of this beam are modulus of elasticity *E*, cross-section moment of inertia *I*, mass density ρ, cross-section area *A* and the coefficient of strain-rate damping $c_{s}$; a proportional damping is assumed. The equation of the forced vibrations of each span (bay) $x_{i}$ (i=1,...,n, in which *n* is the number of parts) is:

(1) $$EI\;\frac{\partial^{4}w_{rel}\left(x_{i},t\right)}{\partial x_{i}^{4}}+c_{s}I\;\frac{\partial^{5}w_{rel}\left(x_{i},t\right)}{\partial x_{i}^{4}\partial t}+\rho A\;\frac{\partial^{2}w_{rel}\left(x_{i},t\right)}{\partial t^{2}}=-\rho A\;\frac{\partial^{2}w_{b}\left(x_{i},t\right)}{\partial t^{2}}.$$

$w_{b}\left(x_{i},t\right)$ is the transverse displacement of any point along the beam caused by the motion of the supports. In general, the two supports have different displacements; therefore, they cause a translation and rotation of the un-deformed beam [9,19]. $w_{rel}\left(x_{i},t\right)$ is the transverse motion of any point of the beam caused by elastic deformation (i.e., the relative motion with respect to the un-deformed beam). In Equation (1), the backward piezoelectric coupling term [9,19] is neglected, because the piezoelectric patches are much thinner and less stiff than the slat skin and because the length of the patches is much smaller than the total length of the slat. Free un-damped vibrations are described by the following homogeneous equation:

(2) $$EI\;\frac{\partial^{4}w_{rel}\left(x_{i},t\right)}{\partial x_{i}^{4}}+\rho A\;\frac{\partial^{2}w_{rel}\left(x_{i},t\right)}{\partial t^{2}}=0.$$

The natural frequencies and the modes of vibration are calculated with the method proposed in [20,21]. The continuous beam is divided into three parts with different coordinate systems and origins, see Figure 3. The deformed shape of the *i*th part of the beam, see Figure 3, is described by the function $\psi_{i}\left(x_{i}\right)$:

(3) $$\psi_{i}\left(x_{i}\right)=A_{i}\;\cosh\left(\gamma x_{i}\right)+B_{i}\;\sinh\left(\gamma x_{i}\right)+C_{i}\;\cos\left(\gamma x_{i}\right)+D_{i}\;\sin\left(\gamma x_{i}\right),$$

in which $A_{i}$, $B_{i}$, $C_{i}$ and $D_{i}$ are unknown constants and parameter γ includes the unknown natural frequency *f*:

(4) $$\gamma =\sqrt[{4}]{{\left(2\pi f\right)}^{2}\cdot \frac{\rho A}{EI}}.$$

This approach leads to (4·n+1) unknowns, which can be found setting (4·n) boundary conditions and solving the eigenvalue problem. In the present case, n=3 and 4 boundary conditions correspond to zero bending moment and shear force at the free ends:

(5) $$-EI\;\frac{\partial^{2}\psi_{1}\left(x_{1}\right)}{\partial x_{1}^{2}}{|}_{x_{1}=0}=0,$$

(6) $$-EI\;\frac{\partial^{3}\psi_{1}\left(x_{1}\right)}{\partial x_{1}^{3}}{|}_{x_{1}=0}=0,$$

(7) $$-EI\;\frac{\partial^{2}\psi_{3}\left(x_{3}\right)}{\partial x_{3}^{2}}{|}_{x_{3}=L_{3}}=0,$$

(8) $$-EI\;\frac{\partial^{3}\psi_{3}\left(x_{3}\right)}{\partial x_{3}^{3}}{|}_{x_{3}=L_{3}}=0.$$

The other four boundary conditions correspond to zero deflections at the supports:

(9) $$\psi_{1}\left(x_{1}\right){|}_{x_{1}=L_{1}}=0,$$

(10) $$\psi_{2}\left(x_{2}\right){|}_{x_{2}=0}=0,$$

(11) $$\psi_{2}\left(x_{2}\right){|}_{x_{2}=L_{2}}=0,$$

(12) $$\psi_{3}\left(x_{3}\right){|}_{x_{3}=0}=0.$$

The four left equations are obtained by imposing the continuity of beam slope and curvature at the supports:

(13) $$\frac{\partial \psi_{1}\left(x_{1}\right)}{\partial x_{1}}{|}_{x_{1}=L_{1}}=\frac{\partial \psi_{2}\left(x_{2}\right)}{\partial x_{2}}{|}_{x_{2}=0},$$

(14) $$\frac{\partial^{2}\psi_{1}\left(x_{1}\right)}{\partial x_{1}^{2}}{|}_{x_{1}=L_{1}}=\frac{\partial^{2}\psi_{2}\left(x_{2}\right)}{\partial x_{2}^{2}}{|}_{x_{2}=0},$$

(15) $$\frac{\partial \psi_{2}\left(x_{2}\right)}{\partial x_{2}}{|}_{x_{2}=L_{2}}=\frac{\partial \psi_{3}\left(x_{3}\right)}{\partial x_{3}}{|}_{x_{3}=0},$$

(16) $$\frac{\partial^{2}\psi_{2}\left(x_{2}\right)}{\partial x_{2}^{2}}{|}_{x_{2}=L_{2}}=\frac{\partial^{2}\psi_{3}\left(x_{3}\right)}{\partial x_{3}^{2}}{|}_{x_{3}=0}.$$

The 4·n boundary conditions can be arranged in a homogeneous linear system:

(17) $$\left[A\right]\left\{v\right\}=0,$$

in which vector $\left\{v\right\}$ contains the unknown constants: $A_{i}$, $B_{i}$, $C_{i}$ and $D_{i}$, i=1, …, *n*. Matrix $\left[A\right]$ depends only on parameter γ. Non-trivial solutions are obtained only if:

(18) $$det\left(\left[A\right]\right)=0.$$

This equation is satisfied only when the parameter γ assumes a set of infinite discrete values $\gamma_{k}$ (k=1,2, …, +∞). The corresponding *k*th natural frequency of the beam can be calculated as follows:

(19) $$f_{k}=\frac{\gamma_{k}^{2}}{2\pi }\cdot \sqrt{\frac{EI}{\rho A}}.$$

The non-null vector $\left\{v_{k}\right\}$ corresponding to $\gamma_{k}$ contains the coefficients of $\psi_{i}\left(x_{i}\right),$i=1,…,3 that define the *k*th mode of vibration of the whole beam $\phi_{k}^{*}\left(x\right)$:

(20) $$\phi_{k}^{*}\left(x\right)=\left\{\begin{array}{cc} \phi_{1,k}^{*}\left(x\right) & if\;0\leq x\leq L_{1}\\ \phi_{2,k}^{*}\left(x-L_{1}\right) & if\;L_{1}\leq x\leq L_{2}\\ \phi_{3,k}^{*}\left(x-L_{1}-L_{2}\right) & if\;L_{2}\leq x\leq L_{3} \end{array}\right.$$

with

(21) $$\phi_{i,k}^{*}\left(x_{i}\right)=A_{i,k}\;\cosh\left(\gamma_{k}x_{i}\right)+B_{i,k}\;\sinh\left(\gamma_{k}x_{i}\right)+C_{i,k}\;\cos\left(\gamma_{k}x_{i}\right)+D_{i,k}\;\sin\left(\gamma_{k}x_{i}\right).$$

**Figure 4:**
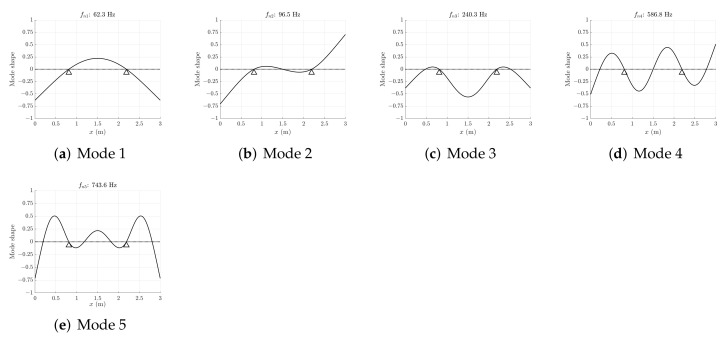
First five modes of vibration of the slat.

Finally, the modes of vibration are normalized with respect to the mass of the beam by means of the equation:

(22) $$\begin{array}{cc} \int_{0}^{L}\rho A\;\phi_{k}^{2}\left(x\right)\;dx=1, & k=1,2,...,+\infty , \end{array}$$

where $\phi_{k}\left(x\right)$ is the mass-normalized mode, which is proportional to the non-normalized mode. Figure 4 shows the first modes of vibration of the slat and the corresponding natural frequencies. The first mode (fundamental mode) has a natural frequency of 62.3 Hz, whereas the fifth mode reaches a natural frequency of 743.6 Hz. The odd modes are symmetric with respect to the slat center, whereas the even modes are anti-symmetric.

### 2.2. Load Conditions

**Figure 5:**
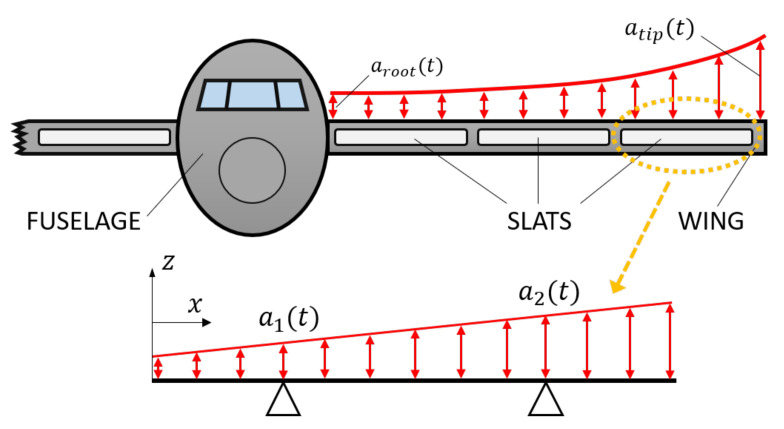
Expected trend of the acceleration along a wing with slats. The distribution of the acceleration along the single slat is a linear function of the *x* coordinate.

A scheme of a wing equipped with slats is depicted in Figure 5. The slat is mounted on the wing, and vibration acceleration amplitude increases from the root of the wing to the tip.

**Figure 6:**
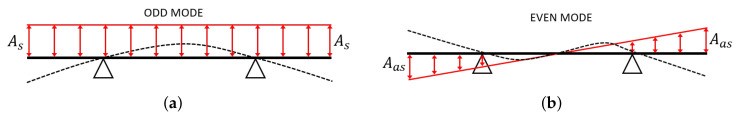
Components of acceleration amplitude: (**a**) symmetric; (**b**) anti-symmetric.

The different acceleration amplitudes of the two supports of the slat $A_{1}$ and $A_{2}$ generate a trapezoidal distribution of acceleration amplitude along the slat and acceleration amplitude $A_{1}$ can be expressed as a function of $A_{2}$:

(23) $$A_{1}=\alpha \cdot A_{2},$$

in which α is a geometric coefficient. The corresponding distribution of inertia forces is the load that excites the slat. The decomposition of the trapezoidal acceleration amplitude distribution into symmetric and anti-symmetric components (Figure 6 is very useful in relation with the excitation of the various modes of vibration of the slat.

The constant amplitude of the symmetric component is given by:

(24) $$A_{s}=A_{2}\cdot C_{s}=\frac{A_{2}}{2}\left[\alpha +1+\left(1-\alpha \right)\cdot \frac{L_{3}-L_{1}}{L_{2}}\right].$$

The maximum amplitude (at the free ends) of the anti-symmetric component is given by:

(25) $$A_{as}=A_{2}\cdot C_{as}=\frac{A_{2}}{2}\left[1-\alpha +\left(1-\alpha \right)\cdot \frac{L_{3}+L_{1}}{L_{2}}\right].$$

**Figure 7:**
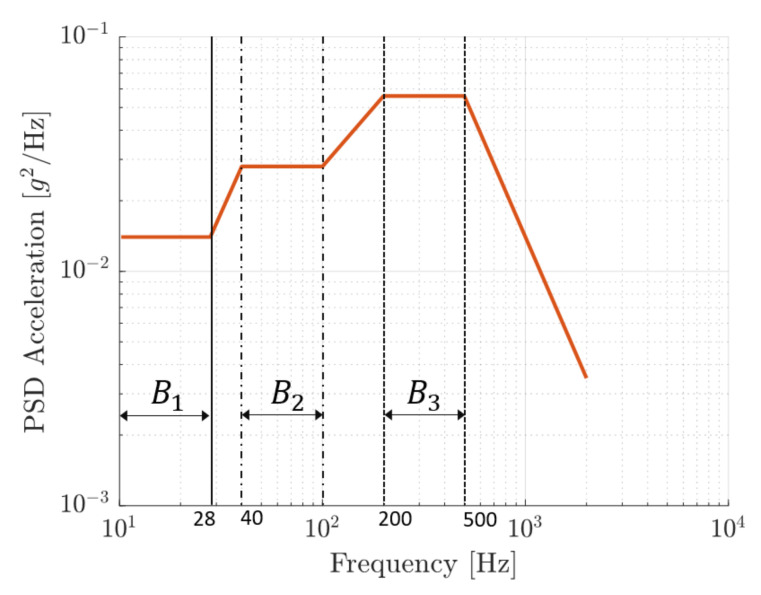
PSD of the acceleration based on the standard specification RTCA-DO-160 CAT S curve E [22]. Three characteristic frequency bands are defined and highlighted.

The parameters $C_{s}$ and $C_{as}$ are geometric constants. One of the main features of this application is that reference acceleration $a_{2}\left(t\right)$ is not a deterministic function of time, but it is a random acceleration defined in the frequency domain by means of its PSD. This PSD is based on the standard specification RTCA-DO-160 CAT S curve E, which is used for testing wing components [22]. It consists in a random acceleration with 10–2000 Hz frequency band and RMS value 7.94 g; see Figure 7.

Since this cycle corresponds to nominal maximum operating conditions, a coefficient of 0.7 was applied to each vibration level. This factor will make the results closer to the real scenario encountered during the aircraft flight.

### 2.3. Frequency Response Functions

The equation of the forced vibrations is solved with the mode superposition method [21,23]. The displacement of any point of the slat due to slat deformation is expressed as a linear combination of the modes of vibration:

(26) $$w_{rel}\left(x,t\right)=\sum_{k=1}^{+\infty }\phi_{k}\left(x\right)\;\eta_{k}\left(t\right),$$

in which $\eta_{k}\left(t\right)$ is the *k*th modal coordinate and $\phi_{k}\left(x\right)$ is the *k*th mass-normalized mode of vibration. Therefore, the equation of the *k*th modal coordinate takes the form:

(27) $$\begin{array}{c} {\ddot{\eta }}_{k}\left(t\right)+2\zeta_{k}\omega_{k}{\dot{\eta }}_{k}\left(t\right)+\omega_{k}^{2}\eta_{k}\left(t\right)=\\ -{\left[\left(\rho A\int_{0}^{L}\phi_{k}\left(x\right)\;dx\right)\cdot a_{2}\left(t\right)\;C_{s}\right]}^{\frac{1-{\left(-1\right)}^{k}}{2}}-{\left[\left(\rho A\int_{0}^{L}\phi_{k}\left(x\right)\left(\frac{2x}{L}-1\right)\;dx\right)\cdot a_{2}\left(t\right)\;C_{as}\right]}^{\frac{1-{\left(-1\right)}^{k+1}}{2}}, \end{array}$$

where at the left-hand side $\omega_{k}=2\pi f_{k}$ and $\zeta_{k}$ is the damping ratio of the *k*th mode. At the right-hand side, the first term, which corresponds to the symmetric component of slat acceleration, is non-zero only for the odd modes; the second term, which corresponds to the anti-symmetric component of slat acceleration is non-zero only for the even modes of vibration.

The relation between shape of the mode of vibration and the spatial distribution of the load on the structure can be quantified by means of a spatial factor. This factor $Is_{k}$ is the integral at the right-end side of Equation (27):

(28) $$\begin{array}{cc} I_{S_{k}}=\int_{0}^{L}\phi_{k}\left(x\right)\;dx, & k\;odd,\\ I_{S_{k}}=\int_{0}^{L}\phi_{k}\left(x\right)\cdot \left(\frac{2x}{L}-1\right)\;dx, & k\;even. \end{array}$$

The first formula holds for the odd modes, which are excited only by the symmetric part of the load. The second formula holds for the even modes, which are excited only by the anti-symmetric part of the load.

The Frequency Response Function (FRF) between the generic modal coordinate and base excitation can be calculated assuming harmonic excitation and response, i.e.,:

(29) $$\begin{array}{c} a_{2}\left(t\right)=A_{2}\;e^{i\omega t},\\ \eta_{k}\left(t\right)=\eta_{0,k}\;e^{i\omega t}. \end{array}$$

Introducing Equations (28) and (29) in (27) the following result holds:

(30) $$FRF_{\eta_{k}}=\frac{-{\left(\rho AI_{S_{k}}\;C_{s}\right)}^{\frac{1-{\left(-1\right)}^{k}}{2}}-{\left(\rho AI_{S_{k}}\;C_{as}\right)}^{\frac{1-{\left(-1\right)}^{k+1}}{2}}}{\omega_{k}^{2}-\omega^{2}+2i\zeta_{k}\omega_{k}\omega }.$$

By expressing the modal coordinates in Equation (26) as functions of the calculated FRFs, the following equation is obtained:

(31) $$w_{rel}\left(x,t\right)=\sum_{k=1}^{+\infty }\phi_{k}\left(x\right)\;FRF_{\eta_{k}}\left(\omega \right)\;A_{2}\;e^{i\omega t}.$$

The FRF between the displacement $w_{rel}$ in a generic point *x* of the beam and the base acceleration $A_{2}$ is derived from Equation (31) as follows:

(32) $$FRF_{w}\left(x,\omega \right)=\sum_{k=1}^{+\infty }\phi_{k}\left(x\right)\;FRF_{\eta_{k}}\left(\omega \right).$$

**Figure 8:**
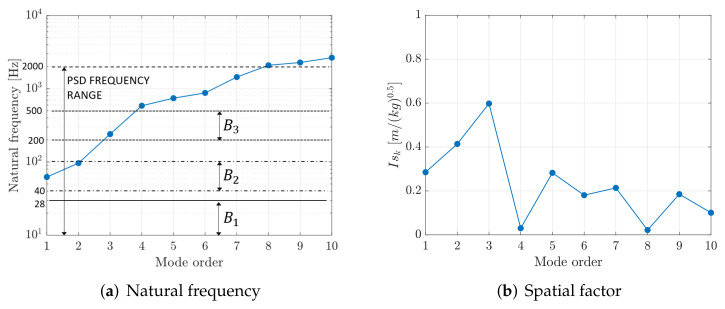
(**a**) Natural frequencies of the slat vs. mode order. The bands of the broad-band excitation from Figure 7 are highlighted. (**b**) Values of the geometric factors $I_{S_{k}}$ vs. mode order.

In practical simulations the summation is truncated, taking into account only the $N_{m}$ modes that are present in the frequency band of interest. Equation (31) shows that the deformed shape of the slat is the result of the contributions of the various modes of vibration, and that $FRF_{\eta_{k}}\left(\omega \right)$ determines the relevance of each modal contribution. $FRF_{\eta_{k}}\left(\omega \right)$ is large when the *k*th mode of vibration is excited at a frequency equal or close to the natural frequency of the mode; in this case, the denominator of Equation (30) is minimum. Figure 8a depicts the natural frequencies of the first ten modes of vibration of the slat in relation with the bands of the broad-band excitation (see Figure 7). It is clear that only the first five modes are significantly excited by the broad-band vibration, whereas the higher-order modes are poorly excited. Figure 8b shows values of factor $Is_{k}$ and highlights that the third vibration mode has the largest matching between the mode shape and the symmetric load distribution, whereas the second mode is the most excited by the anti-symmetric load distribution.

The analysis of the two plots of Figure 8 highlights that the most important modes of the slat are the second and the third, since their natural frequencies belong to the band of excitation and since they have large geometric factors $Is_{k}$.

The strain within the piezoelectric material can be calculated as:

(33) $$S\left(x,t\right)=-h_{c}\cdot \frac{\partial^{2}w_{rel}\left(x,t\right)}{\partial x^{2}},$$

in which $h_{c}$ is the distance of the piezo patch from the neutral axis of the slat cross-section. By letting Equation (31) in (33), the following FRF between the strain and the excitation can be calculated:

(34) $$FRF_{S}\left(x,\omega \right)=-h_{c}\sum_{k=1}^{\infty }\frac{\partial^{2}\phi_{k}\left(x\right)}{\partial x^{2}}FRF_{\eta_{k}}\left(\omega \right).$$

## 3. Harvesting by Means of Piezo-Patches

The PE patch considered in the following numerical analysis is the MFC-8514-P2, manufactured by the Smart Material Corp. The active piezo-layer is made of Macro Fibre Composite (MFC), which shows improved damage tolerance and flexibility with respect to monolithic ceramic materials. The PE patch is assumed to be directly attached to the skin of the slat, in order to exploit the deformation induced by the vibration of the structure.

**Figure 9:**
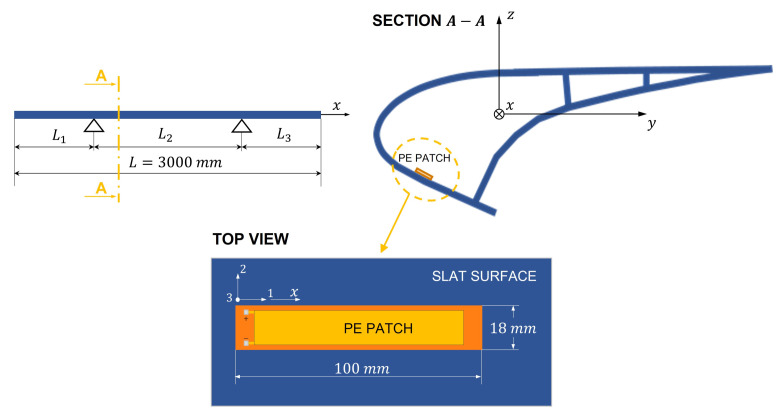
Example of a piezoelectric patch directly attached to the slat’s surface. The local reference frame of the patch has axes (1, 2, 3), whereas the global reference frame has axes (*x*, *y*, *z*).

The sizes of the PE patch are much smaller than the sizes of the slat; hence, there are many positions where the PE patch can be attached to the slat’s surface. The power generated by the patch depends on the position, since the vibration level changes along the slat. The dependence of the generated power on the PE patch position is analyzed in Section 3.2. Figure 9 shows an example of PE patch attached to the slat’s surface and shows the local reference frame of the patch (axes 1, 2, 3) and the global reference frame (axes *x*, *y*, *z*). The patch is designed to exploit the axial strain of the substrate (1-direction) and is polarized in the direction perpendicular to its middle plane (3-direction, piezoelectric effect $d_{31}$). Therefore, the patch is oriented with its 1-axis parallel to the x-axis of the slat reference frame, since the most important deformation of the slat is the axial strain caused by the transverse load distribution. The electro-mechanical parameters of the patch are represented in Table 1.

### 3.1. Calculation of Voltage and Power

It is assumed that the strain within the patch is equal to the strain of the underlying portion of the slat, since the PE patch is very thin and flexible and is directly attached to the skin. In this condition, the Open Circuit Voltage (OCV) generated by the patch is strictly related to the strain of the slat as discussed in [24], that is:

(35) $$v_{oc}\left(t\right)=-\frac{{\bar{e}}_{31}\;b}{C_{p}}\cdot \int_{L_{1}}^{L_{2}}h_{c}\cdot \frac{\partial^{2}w_{rel}\left(x,t\right)}{\partial x^{2}}\;dx,$$

where $L_{1}$ and $L_{2}$ define the positions of the shorter edges of the patch with respect to the reference frame of the slat; *b* is the width of the patch; and ${\bar{e}}_{31}$ is the piezoelectric constant in the hypothesis of plane stress condition. Parameter $C_{p}$ is the capacitance of the piezoelectric patch:

(36) $$C_{p}=\frac{\epsilon_{33}^{S}bL}{h_{p}},$$

where *L* and $h_{p}$ are the length and the thickness of the patch, respectively; $\varepsilon_{33}^{S}$ is the relative permittivity at constant strain.

Function $w_{rel}\left(x,t\right)$ is related to acceleration amplitude $A_{2}$, Equation (31). The relation between the generated OCV ($v_{oc}$) and $A_{2}$ is obtained introducing Equation (31) in (35):

(37) $$v_{oc}\left(t\right)=-\frac{{\bar{e}}_{31}\;b\;h_{c}}{C_{p}}\cdot \sum_{k=1}^{+\infty }\left(\int_{L_{1}}^{L_{2}}\frac{\partial^{2}\phi_{k}\left(x\right)}{\partial x^{2}}\;dx\right)FRF_{\eta_{k}}\left(\omega \right)A_{2}\;e^{i\omega t}.$$

The FRF between $v_{oc}$ and $A_{2}$ is given by:

(38) $$FRF_{v_{oc}}\left(\omega \right)=-\frac{{\bar{e}}_{31}\;b\;h_{c}}{C_{p}}\cdot \sum_{k=1}^{+\infty }\left(\int_{L_{1}}^{L_{2}}\frac{\partial^{2}\phi_{k}\left(x\right)}{\partial x^{2}}\;dx\right)FRF_{\eta_{k}}\left(\omega \right).$$

The generated voltage is a broad-band signal since the base acceleration is represented by a broad-band excitation. Therefore, the $FRF_{v_{oc}}\left(\omega \right)$ is used to determine the Power Spectral Density (PSD) of the open-circuit voltage $PSD_{v_{oc}}\left(\omega \right)$. The $PSD_{v_{oc}}\left(\omega \right)$ is given by:

(39) $$PSD_{v_{oc}}\left(\omega \right)={\left|FRF_{v_{oc}}\left(\omega \right)\right|}^{2}\cdot PSD_{a_{2}}\left(\omega \right).$$

According to the *Parseval’s theorem*, the RMS value of the OCV is calculated from the $PSD_{v_{oc}}\left(\omega \right)$ as follows:

(40) $$V_{RMS}=\sqrt{\int_{0}^{+\infty }PSD_{v_{oc}}\left(\omega \right)\;d\omega }.$$

**Figure 10:**
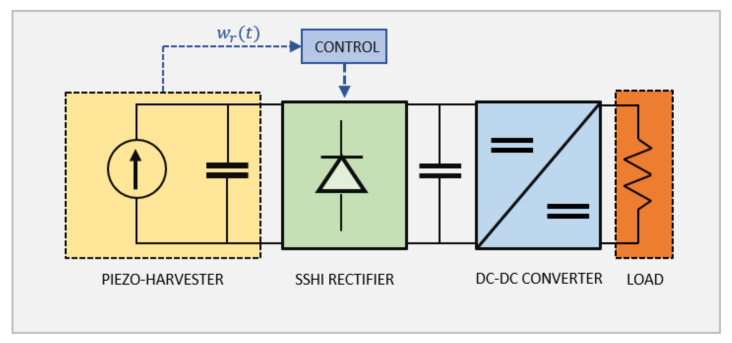
Electrical circuit of the piezoelectric energy generator and the PMU. The piezoelectric harvester is represented as a current generator in parallel with a capacitor [9], whereas the electric load is represented by a resistor.

Piezoelectric energy harvesters provide an alternating voltage output, which is usually characterized by a predominant harmonic component. However, electronic devices typically require a DC voltage input. Therefore, piezoelectric energy generators are equipped with a power management unit (PMU), which uses an AC-DC voltage rectifier and a DC-DC converter for impedance matching with the connected electronic device. The scientific literature provides different PMU schemes for highly efficient energy conversion. The synchronized switch harvesting on inductor (SSHI) technique has shown the most promising results in boosting the performance of the energy generator [25,26]. Figure 10 represents a PMU based on a SSHI rectifier.

The OCV generated by each piezo-patch is a random signal, due to the random excitation applied to the slat. Nevertheless, the larger amount of the electrical energy is produced by the harmonic components corresponding to the most excited vibration mode of the slat structure. In order to estimate the generated power by each piezo-patch, the electrical energy harvested from the random vibration is assumed to be generated by an equivalent harmonic voltage, having the same frequency *f* of the maximum peak in the $FRF_{v_{oc}}$. With this assumption, the maximum harvested energy is given by:

(41) $$W_{max}=\frac{1}{2}C_{p}V_{max}^{2}.$$

$V_{max}$ is the maximum generated voltage that derives from the RMS value as follows:

(42) $$V_{max}=\sqrt{2}\cdot V_{RMS}.$$

Assuming the hypothesis of harmonic voltage signal, the maximum average power generated by an SSHI rectifier is given by:

(43) $$P_{max}=2\;f\;\left(\frac{1+\gamma }{1-\gamma }\right)\;W_{max},$$

where γ is the inversion coefficient and it is a specific feature of the SSHI rectifier. The inversion coefficient theoretically ranges from 0 to 1. The maximum energy is magnified by a factor (1+γ)/(1−γ)≥1, which is the key feature of the SSHI harvesting technique.

### 3.2. Calculated Results

Figure 8 shows that the broad-band excitation is able to stimulate specific modes of vibration characterized by vibration nodes and anti-nodes. Therefore, the dependence of the generated power on patch position has to be analyzed. In order to carry out this analysis, a grid of test positions is defined, which consists of three rows of thirty evenly spaced positions along the slat’s length. The only difference between the three rows is the distance of the PE patch from the neutral axis of the slat’s cross-section.

Specific data about slat damping ratio are not available, but the value of the wing damping ratio in [27] ranges from 0.01 to 0.08. In the framework of this research, a slat damping ratio equal to 0.05 has been chosen because it is equal to the damping ratio of the cantilever beam, which is made of composite material as well (see Section 4). This choice makes easier the comparison between the two analyses.

**Figure 11:**
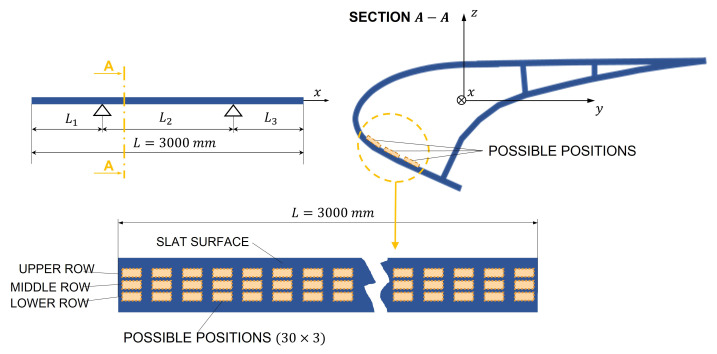
Grid of test positions.

Figure 11 schematizes the grid of test positions.

**Figure 12:**
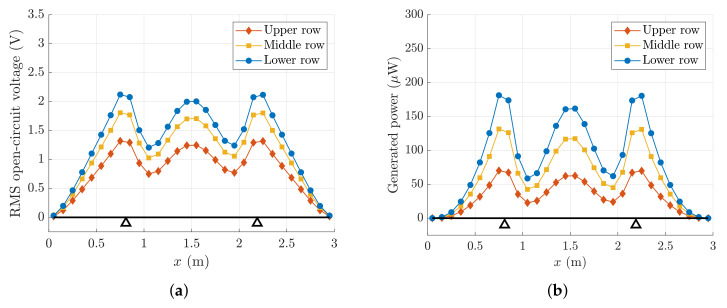
Performance of the PE patch directly attached to the surface of the slat. (**a**) RMS value of the OCV; (**b**) generated power.

Figure 12a,b represent the RMS values of the OCV and the power output generated by a single PE patch, as a function of the position on the slat according to the grid of Figure 11. The voltage and power are calculated by means of Equations (40) and (43), respectively.

Figure 12 shows that the patch has the largest voltage and power output when it is attached in the lower row, since the performance is strictly related to the parameter $h_{c}$, as shown by Equation (35). Moreover, Figure 12 highlights that the performance depends on the location of the patch along the slat. The trends of both the voltage and power are strictly related to the shape of the third mode of vibration of the slat. This mode of vibration has the largest spatial factor and in the frequency band $B_{3}$ is excited in resonance, hence it dominates the vibration of the slat structure. The voltage output decreases to zero at the two ends of the slat, since the curvature is zero.

Since the sizes of the patches are much smaller than the sizes of the slat, several patches can be attached in the best positions to generate more electric power.

### 3.3. Validation by Means of a Finite Element Model

A three-dimensional (3D) finite element (FE) model was developed in the framework of this research. The FE model analyzes the performance of a single patch located in one of the several test positions shown in Figure 11. The patch is directly bonded to an equivalent underlayer, which represents only a small portion of the slat and simulates the behavior of the slat just below the patch. A 3D FE model of the whole slat would require a very large computational effort since many thin elements would be required to discretize the slat structure.

The equivalent underlayer is subjected to the same strain distribution as the one in the analytical model. The FE model is developed in COMSOL, and a frequency domain analysis is performed within the frequency range 10–1000 Hz. The OCV generated by the piezo-patch in the FE model is evaluated, and an FRF between the OCV and the acceleration $A_{2}$ of the support of the slat is calculated.

**Figure 13:**
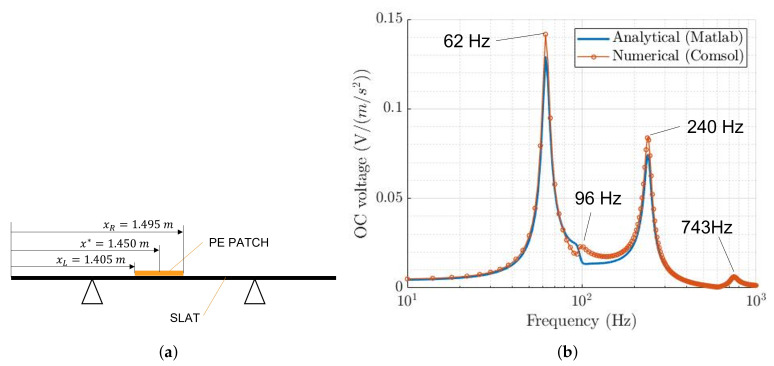
(**a**) Location of the considered PE patch; (**b**) comparison between the analytical and numerical FRFs.

Figure 13a highlights the location of the patch considered in this analysis, whereas Figure 13b compares the FRFs between the OCV and the acceleration of the support, calculated through the analytical model and the FE model in COMSOL.

Figure 13b shows that the analytical and the numerical FRFs are in good agreement. The modes of vibration of the slat and the peak values are correctly described. The RMS value of the OCV generated by the patch in the FE model is estimated using Equations (39) and (40). It is worth noticing that the FRF in Equation (39) is calculated by means of the FE model. Table 2 compares the RMS value of the OCV calculated by means of the analytical and numerical model. There is a good agreement between the two values, and this corroborates the reliability of the analytical model presented in the previous sections.

## 4. Harvesting by Means of Cantilever Harvesters

The PE patches directly attached to the skin of the slat exploit the available vibration of the structure, but they cannot be tuned to a specific frequency, in order to optimize their performance. On the contrary, PE cantilever harvesters can be tuned to the frequency of the broad-band excitation which shows the largest acceleration level, exploiting the resonance phenomenon. This section aims to analyze the performance of cantilever harvesters and compare the results with the performance of the piezoelectric patches attached to the skin.

### 4.1. Cantilever Model

**Figure 14:**
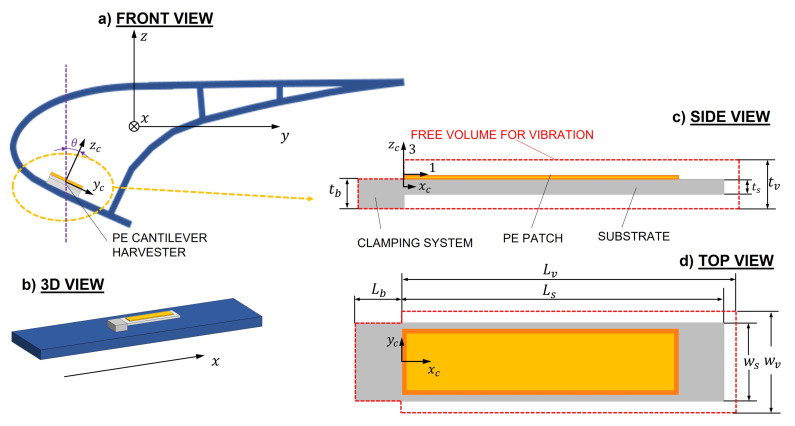
Example of PE cantilever harvester: (**a**) arrangement of the cantilever harvester; (**b**) the 3D view; (**c**) side view of the cantilever harvester and required free volume for vibration; (**d**) top view of cantilever harvester.

The cantilever harvester consists of a single piezoelectric layer bonded to a cantilever beam. The cantilever vibrates in the transverse direction along the $z_{c}$-axis of the local reference frame. This axis is rotated by an angle θ with respect to the z-axis of the global reference frame of the slat. Figure 14 shows an example of PE cantilever harvester mounted on the slat and highlights the orientation of the harvester attached to the slat. Figure 14 also highlights the local reference frames of the cantilever beam (axes $x_{c}$, $y_{c}$, $z_{c}$) and the piezoelectric patch (axes 1, 2, 3) and the global reference frame of the slat (axes *x*, *y*, *z*).

The 1-axis is parallel to the $x_{c}$-axis of the local reference frame of the cantilever.

The piezoelectric layer is the same piezoelectric patch that in Section 3 was directly attached to the skin of the slat (Smart Material MFC-8514-P2). The substrate consists of a glass-reinforced epoxy laminate material (FR-4); the geometrical and mechanical properties of the cantilever harvester are summarized in Table 3.

**Figure 15:**
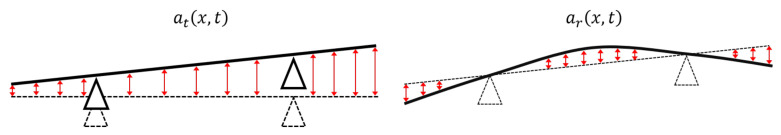
Components of the total base acceleration which excites the PE cantilever beam.

The cantilever harvester is modeled with a single-mode approach, which in most cases gives accurate results [9] and is widely described in [19,24] and validated in [28]. However, the base acceleration term a(x,t), which excites the cantilever beam, must be analyzed and discussed. The base acceleration *a* can be defined as follows:

(44) $$a\left(x,t\right)=cos\left(\theta \right)\cdot [a_{t}\left(x,t\right)+a_{r}\left(x,t\right)],$$

where θ is the angle between the $z_{c}$-axis of the cantilever reference frame and the z-axis of the slat reference frame, as represented in Figure 14, $a_{t}$ is the acceleration component due to the rigid motion of the slat and $a_{r}$ is the contribution due to the deformation of the slat; see Figure 15.

The base acceleration due to the rigid motion is the following:

(45) $$a_{t}\left(x,t\right)=\left[\frac{\alpha }{L_{2}}\left(L_{1}+L_{2}-x\right)+\frac{1}{L_{2}}\left(x-L_{1}\right)\right]\cdot A_{2}\;e^{i\omega t}.$$

The acceleration due to the deformation of the slat is the second derivative of the displacement function $w_{rel}\left(x,t\right)$ in Equation (31), and it is defined as follows:

(46) $$a_{r}\left(x,t\right)=\frac{\partial^{2}w_{rel}\left(x,t\right)}{\partial t^{2}}=-\omega^{2}\cdot FRF_{w}\left(x,\omega \right)\cdot A_{2}\;e^{i\omega t}.$$

The extended formula of the base acceleration is calculated introducing Equations (45) and (46) in (44):

(47) $$a\left(x,t\right)=cos\left(\theta \right)\cdot \left[\frac{\alpha }{L_{2}}\left(L_{1}+L_{2}-x\right)+\frac{1}{L_{2}}\left(x-L_{1}\right)-\omega^{2}FRF_{w}\left(x,\omega \right)\right]\cdot A_{2}\;e^{i\omega t},$$

(48) $$a\left(x,t\right)=C_{a}\left(x,\omega \right)\cdot A_{2}\;e^{i\omega t},$$

in which function $C_{a}$ depends on space and frequency.

The FRF between the OCV generated by the cantilever harvester and the base acceleration is given by the following equation:

(49) $$FRF_{v_{oc}}\left(\omega ,x\right)=\frac{\varphi_{1}}{C_{p}}\cdot \frac{-\int_{0}^{L}m_{c}\;\phi_{1}\left(x_{c}\right)\;dx_{c}}{\omega_{1}^{2}-\omega^{2}+2\;i\;\zeta_{1}\;\omega_{1}\;\omega +\frac{\chi_{1}\varphi_{1}}{C_{p}}}\cdot C_{a}\left(x,\omega \right),$$

where $\varphi_{1}$ is the electro-mechanical modal coupling term, $\chi_{1}$ is the backward electromechanical modal coupling term, $m_{c}$ is the mass per unit length of the PE cantilever, $\phi_{1}$ is the first mass-normalized vibration mode of the cantilever and $\omega_{1}$, $\zeta_{1}$ are the natural frequency and the damping ratio of the first vibration mode of the cantilever. It is worth noticing that the coordinate $x_{c}$ defines a point along the cantilever beam, whereas the coordinate *x* identifies a point along the slat.

The RMS value of the OCV generated by the piezo-cantilever is given by Equations (39) and (40).

Strain FRF can be calculated with an equation similar to Equation (34), in which the mode of the cantilever $\phi_{1}\left(x_{c}\right)$ is used and $h_{c}$ is the distance from the neutral axis of the cantilever cross-section.

### 4.2. Calculated Results

**Figure 16:**
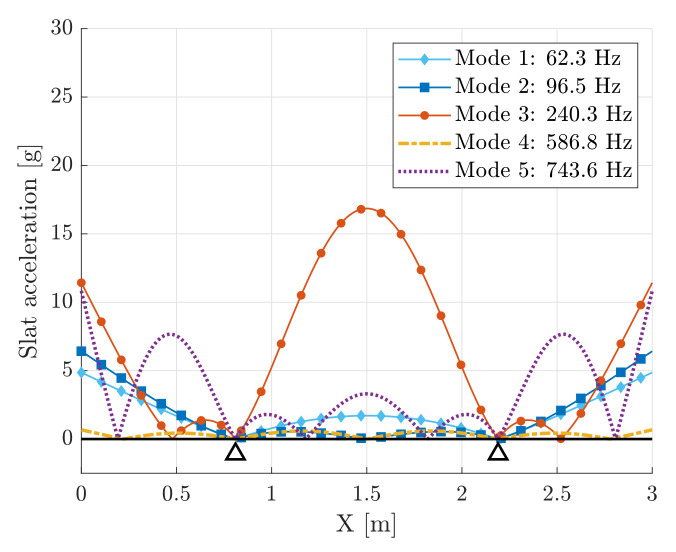
RMS values of the acceleration at any point of the slat due to the single mode of the slat with a trapezoidal load distribution.

Equation (47) highlights that the base acceleration of the cantilever is strictly related to the vibration modes of the slat. Hence, the analysis of the acceleration level related to each vibration mode provides a guideline to maximize the performance of the cantilever harvester. Figure 16 represents the contribution of each vibration mode of the slat to the global acceleration level. Only the first five modes are considered. The distribution of the force of inertia acting on the slat is assumed trapezoidal with α=0.5.

Figure 16 highlights that the third mode of the slat provides the largest excitation level with a maximum located at the middle point of the slat length. Indeed, the third mode is excited in the frequency band $B_{3}$ and has the largest value of the geometric factor $I_{S_{k}}$; see Figure 8a,b.

In order to highlight the effectiveness of tuning, two PE cantilever harvesters tuned to the second and third modes of the slat are considered. The natural frequency of the cantilever harvester is changed acting on the thickness $t_{s}$ of the substrate, whereas the other dimensions are kept constant as in Table 3. The thickness $t_{s}$ and the corresponding natural frequency of the cantilever are summarized in Table 4. The frequency band of the acceleration PSD which corresponds to the natural frequency of the cantilever is highlighted as well.

In order to highlight the dependence of the power generated by the PE cantilever on the position along the slat, a row of 30 test positions is considered.

**Figure 17:**
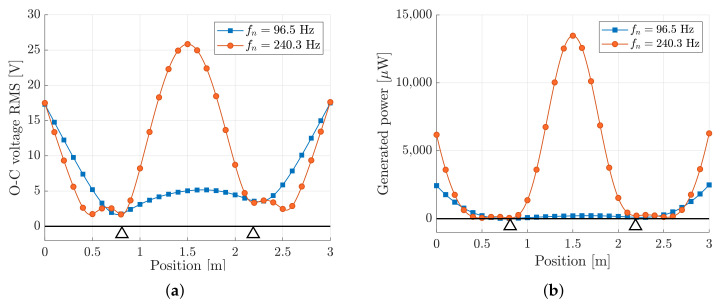
Performance of the piezo-cantilever harvester as a function of tuning and position: (**a**) RMS values of the generated OCV; (**b**) generated power.

Figure 17 shows that the harvester #2 generates much more voltage and power than harvester #1, since the third mode of the slat is strongly excited. It is worth noticing that the maximum power generated by the two PE cantilever harvesters is much larger that the power generated by the PE patches directly attached to the slat, as shown in Figure 12. The resonance phenomenon, which can be exploited tuning a cantilever harvester, allows to boost the performance of the energy generator.

The position of the PE cantilever is important as well. Actually, the best locations are at the center of the slat, which is a vibration anti-node of the third mode of vibration.

Since the sizes of the PE cantilever are much smaller than the sizes of the slat, several PE cantilevers can be mounted in the best positions to generate more electric power.

## 5. Discussion

Both the PE patch (strain harvester) and the PE cantilever harvester are based on the same piezoelectric layer and can be equipped with the same PMU. Therefore, a comparison between the two designs in terms of volume and mass of the bare harvester is meaningful. In the cantilever harvester the mass and volume of the structural substrate and the mass of the small block that is used for clamping the cantilever to the slat surface have to be considered as well. The cantilever volume has to take into account that a clearance is needed to make possible the vibrations of the cantilever tip without interference with the slat surface. In the best location and in resonance the maximum tip displacement is $0.89\cdot 10^{-3}\;m$, but a larger free volume for vibration has to be designed, to take into account the possibility of sudden acceleration peaks and to simplify the manufacturing process. Both the piezo patch and the cantilever harvester can be bonded to the vibrating surface by means of structural adhesives.

The PE patch attached in the best position (close to the support) is compared with the PE cantilever with the best tuning and mounted in the best position (at the center).

Results are summarized in Table 5 and show that the mass and especially the volume of the cantilever harvester are larger than the ones of the piezo patch.

Therefore, the power density $P_{d}$ (which is the power-to-volume ratio) of the cantilever harvester is only 30% larger than the one of the piezo patch. The specific power $P_{m}$ (which is the power-to-mass ratio) of the cantilever harvester is about four times larger than the one of the piezo patch.

Finally, it is worth noticing that a cantilever harvester that generates a large voltage is a highly stressed component [29]. Large variable stress may lead to fatigue failure of the piezoelectric layer, with a degradation of piezoelectric properties [30]. The typical lifetime of MFC patches is $10^{10}$ cycles with a strain level lower than 600 ppm [31]. Table 5 clearly shows that the piezo patch will operate well below the strain limit that guarantees the lifetime, whereas the cantilever harvester will be strained slightly above the strain limit with a reduction of the lifetime.

## 6. Conclusions

A semi-analytical mathematical model capable of simulating the performance of a strain harvester (PE patch) attached to a slat has been developed and validated. This model has made it possible to find the best position of the strain harvester along the slat. The performance of the strain harvester (attached in the best position) has been compared with the performance of a tuned cantilever harvester (mounted in the best position).

The simulations have shown that potentially a cantilever harvester, which exploits the resonance phenomenon, is much more effective than a strain harvester, but the requested volume and mass may hinder the implementation of this device in the aeronautical environment, where mass, room and safety requirements are very stringent. Moreover, the integration of the strain harvester with an existing structure is simpler and a better exploitation of the whole vibrating surface is possible, since the strain harvester is more compact.

## Figures and Tables

**Table 1:** Electromechanical properties of the PE patch MFC-8514-P2, from datasheet provided by Smart Material Corp.

Parameter	Unit	Value
Overall length	mm	100
Active length	mm	85
Overall width	mm	18
Active width	mm	14
Thickness	mm	0.3
Young’s modulus at constant electric field in 1 direction	GPa	30.34
Density	kg/m${}^{3}$	5440
Piezoelectric strain constant	pC/N	−210
Capacitance	nF	84.04

**Table 2:** RMS values of the OCV calculated by means of the analytical and numerical models.

Approach	$V_{\mathrm{oc}}$ RMS (V)
Analytical	1.243
Numerical	1.397

**Table 3:** Geometrical and mechanical parameters of the piezoelectric cantilever harvester.

Parameter	Unit	Value
$L_{s}$	mm	120
$w_{s}$	mm	18
Young’s modulus	GPa	24
Density	kg/m${}^{3}$	1850
Damping ratio	/	0.05
$L_{b}$	mm	30
$t_{b}$	mm	7.7
$L_{v}$	mm	122
$w_{v}$	mm	22
$t_{v}$	mm	10
θ	deg	32

**Table 4:** Thickness and natural frequency of the cantilever harevsters.

N.	Thickness $t_{s}$ (mm)	Natural Frequency (Hz)	Frequency Band
#1	2.2	96.5	B2
#2	5.7	240.3	B3

**Table 5:** Mass and volume of the piezo-cantilevers and piezo patches.

Type	Tot. Mass	Tot. Volume	Max Power Density	Max Specific Power	Max Strain
	(kg)	($m^{3}$)	$P_{d}$ (μW/$m^{3}$)	$P_{m}$ (μW/kg)	
Patch	0.002	$5.40\times 10^{-7}$	$3.36\times 10^{8}$	$9.07\times 10^{4}$	$25\times 10^{-6}$
Cantilever #2	0.0325	$3.10\times 10^{-5}$	$4.35\times 10^{8}$	$41.45\times 10^{4}$	$659\times 10^{-6}$

## Data Availability

Not applicable.
